# Evaluating the Efficacy of High-Purity Type I Collagen-Based Skin Substitute Versus Dehydrated Human Amnion/Chorion Membrane in the Treatment of Venous Leg Ulcers: A Randomized Controlled Clinical Trial

**DOI:** 10.7759/cureus.89031

**Published:** 2025-07-30

**Authors:** Naveen Narayan, Chethan Shivannaiah, Suhas Gowda

**Affiliations:** 1 Plastic Reconstructive and Aesthetic Surgery, Adichunchanagiri Institute of Medical Sciences, B G Nagara, IND

**Keywords:** chronic leg ulcers, dehydrated human amnion/chorion membrane, dhacm, helicoll®, high-purity type i collagen-based skin substitute, hptc, venous insufficiency, venous leg ulcers, wound healing

## Abstract

Background

Venous leg ulcers (VLUs) are chronic, difficult-to-heal wounds caused by venous insufficiency that significantly impact patient quality of life. Current treatment options often include compression therapy, wound debridement, and advanced dressings. Advanced wound care products such as high-purity type I collagen-based skin substitutes (HPTCs) and dehydrated human amnion/chorion membrane (dHACM) have emerged as promising therapeutic options. This randomized, controlled clinical trial aimed to compare the clinical efficacy and healing outcomes of HPTC versus dHACM in the treatment of VLUs.

Methodology

This prospective, randomized, controlled study was conducted at a tertiary care hospital. A total of 60 patients with chronic VLUs were randomized into the following two groups: Group A received HPTC (n = 30), and Group B received dHACM (n = 30). Patient demographics, ulcer characteristics, pain scores, and healing rates were recorded over a six-week period. Percentage wound size reduction and vascular infiltration were primary outcomes. Time taken for complete healing, pain reduction, quality of life improvement, recurrence, scar quality, and adverse events were secondary outcomes.

Results

Complete wound closure was achieved in 70% (21/30) of HPTC-treated patients versus 43.3% (13/30) of dHACM-treated patients (p < 0.05). The mean time to complete healing was significantly shorter in the HPTC group (42.6 ± 9.8 days) compared to the dHACM group (46.2 ± 8.7 days, p = 0.047). The mean percentage wound closure at seven weeks was 78.9 ± 17.8 % for HPTC versus 65.4 ± 7.9 % for dHACM (p < 0.001). The comprehensive histopathological analysis at day five post-application provided statistically significant improvements in vascularity infiltration (46% increase), neo-epithelialization (64% increase in migration), fibroblast activity (45% increase), capillary density (65% increase), optimal inflammatory modulation (43% reduction in acute inflammation), and superior collagen deposition (49% increase) favoring HPTC. Pain scores showed significant improvement in both groups. Adverse events were minimal in both groups. The structural stability of the scars was better rated in the HPTC group. No significant difference in recurrence rate was observed.

Conclusions

HPTC demonstrated superior efficacy over dHACM in treating VLUs, with faster healing rates, higher closure percentages, pain reduction, and scar quality in VLUs, supporting its role as a preferred advanced skin substitute and as an effective treatment option for chronic VLUs.

## Introduction

Venous leg ulcers (VLUs) are the most common type of chronic lower extremity ulcerations, accounting for 60-80% of all leg ulcers [1]. They affect approximately 0.18% to 1.35% of the general population and impose a substantial economic burden on healthcare systems worldwide [2]. Chronic venous insufficiency leads to sustained hypertension in the venous system, resulting in capillary damage and impaired healing [3]. These ulcers present a significant public health challenge, leading to impaired quality of life, substantial healthcare expenditures, and recurrence rates exceeding 70% [4]. Standard treatments include compression therapy and conventional wound dressings; however, healing rates remain suboptimal, often resulting in prolonged healing times and high recurrence rates [5].

The pathophysiology of VLUs involves complex interactions among venous hypertension, inflammatory processes, and impaired cellular function within the wound environment [6]. Chronic venous insufficiency leads to increased hydrostatic pressure, capillary leakage, and tissue hypoxia, creating a hostile microenvironment that impedes normal wound healing processes [7,8]. The presence of elevated matrix metalloproteinases, chronic inflammation, and bacterial colonization further contributes to delayed healing and wound chronicity [9].

Wound healing is a complex biological process involving phases of inflammation, proliferation, and remodelling. Traditional wound care often fails to adequately stimulate this cascade in chronic wounds, necessitating the use of advanced biologic dressings [10]. Recent advances in wound care have led to the development of bioengineered skin substitutes designed to address the underlying molecular and cellular deficits in chronic wounds [11]. Advanced wound care options such as collagen-based skin substitutes and allografts have been developed to enhance the wound healing microenvironment. Among these, type I collagen-based skin substitutes (HPTCs) (such as Helicoll®) and dehydrated human amnion/chorion membrane (dHACM) have gained prominence [12-15].

HPTC is a high-purity type I collagen matrix derived from bovine skin and processed to preserve native triple-helix structure, providing a biocompatible scaffold for cellular ingrowth and promoting angiogenesis [16]. dHACM comprises preserved amniotic tissue layers, rich in cytokines and growth factors, and extracellular matrix components that theoretically promote wound healing through regenerative mechanisms.

Clinical studies have demonstrated the efficacy of dHCAM in various chronic wound types [17]. Recent systematic reviews indicate that bioengineered skin substitutes may improve healing outcomes in VLUs, with healing probabilities ranging from 0.11 to 0.65 over variable periods. Clinical trials with dHACM have demonstrated healing rates of 75% at 12 weeks in VLUs [18-20]. Prior studies in diabetic foot ulcers have reported promising results using HPTC compared to dHACM [21,22]. While numerous studies have evaluated treatments individually, no direct comparative studies have evaluated their relative efficacy in VLU management. The present study aims to bridge this gap by conducting a randomized, controlled clinical trial comparing the efficacy of HPTC and dHACM in terms of healing efficacy and patient-reported outcomes.

## Materials and methods

This single-center, prospective, randomized, open-label, single-blinded, controlled, parallel-group clinical trial was conducted at a tertiary care center over a six-month period. The study protocol was reviewed and approved by the Institutional Ethics Committee. All participants provided written informed consent before enrolment. Additionally, all study products used in this study were manufactured, handled, and stored in accordance with applicable Good Manufacturing Practices.

Inclusion and exclusion criteria

In total, 60 patients aged 18-80 years with clinically and duplex ultrasound-diagnosed non-infected chronic venous ulcers due to superficial chronic venous insufficiency, of more than four weeks’ duration, measuring between 2 cm^2^ and 25 cm^2^, with adequate arterial inflow (Ankle Brachial Index >0.8) and willingness to comply with follow-up were included. Exclusion criteria included active infection requiring systemic antibiotics, uncontrolled diabetes, peripheral arterial disease, autoimmune disorders, malignancy, immunosuppression, pregnancy, known allergies to bovine or human-derived products, and those requiring surgical intervention for venous insufficiency. Patients were randomly allocated to two groups using a computer-generated sequence. Group A was treated with HPTC (n = 30), and Group B was treated with dHACM (n = 30).

Intervention

Application protocols followed the manufacturer’s instructions. Wounds were debrided before initial grafting. All patients received standard wound cleaning and compression therapy. In addition, Group A received application of HPTC, a high-purity type I collagen dressing. The product was sized to cover the entire wound surface with a 1 cm margin and secured with appropriate secondary dressings. Group B received a dHACM graft. The membrane was rehydrated and applied to completely cover the wound bed. Standard wound care for both groups involved a first layer of a non-adherent and porous paraffin gauze, followed by a second layer of absorbent gauze pads, and, finally, a third layer of a soft roll and crepe bandage. Infection control measures were followed, and patients were educated regarding wound care and leg elevation. Repeat application was done if deemed necessary.

Outcome measures

The primary outcome measure was percentage wound area reduction, i.e., percentage wound area reduction from week one through week six, as well as one-week follow-up, measured manually with digital photography. Secondary outcome measures were infiltration of vascularity in the ulcer bed, time to achieve complete wound closure, proportion of patients to obtain complete closure, mean number of repeated applications, and incidence of adverse events.

Vascularity assessment was done using biopsy on day zero of application to be compared with day five after the application of HPTC or dHCAM (time frame = six days). For histopathological assessment, before application and on the fifth day after the application of either HPTC or dHCAM, a 2 mm punch biopsy was obtained from the wound edge extending into the wound bed under local anesthesia (2% lidocaine without epinephrine). Biopsy samples were immediately fixed in 10% neutral buffered formalin for 24 hours, processed through graded alcohol, and embedded in paraffin blocks. Serial sections of 4 μm thickness were prepared and stained with hematoxylin and eosin (H&E) for general morphology, Masson’s trichrome for collagen assessment, CD31 immunohistochemistry for capillary density evaluation, and α-SMA immunohistochemistry for fibroblast activity. Histological parameters that were evaluated included vascular infiltration, neo-epithelialization, fibroblast activity, capillary density, inflammatory response, and collagen deposition (Table 1). All histological assessments were performed by two independent pathologists blinded to treatment allocation. Inter-observer agreement was assessed using Cohen’s kappa coefficient.

**Table 1:** Histological parameters evaluated to assess infiltration of vascularity in the ulcer bed on day five of application.

Parameter	Measurement tool	Criteria	Score
Vascular infiltration	Assessed by counting new blood vessels (0–3 scale)	Minimal vascular ingrowth (<5 vessels/HPF)	0
		Mild infiltration (5–10 vessels/HPF)	1
		Moderate infiltration (11–20 vessels/HPF)	2
		Abundant infiltration (>20 vessels/HPF)	3
Neo-epithelialization	Measured as epithelial migration distance from wound edge (0–3 scale)	No epithelial migration	0
		Minimal migration (<25% wound coverage)	1
		Moderate migration (25–75% coverage)	2
		Extensive migration (>75% coverage)	3
Fibroblast activity	Quantified by counting α-SMA positive fibroblasts per HPF and assessment of fibroblast morphology (0–3 scale)	Sparse, inactive fibroblasts	0
		Moderate cellularity, minimal matrix production	1
		High cellularity, active-matrix synthesis	2
		Very high activity with extensive matrix deposition	3
Capillary density	Evaluated using CD31 staining, counted as vessels per mm² of tissue		
Inflammatory response	Graded semi-quantitatively (0–3 scale)	Minimal inflammatory infiltrate	0
		Mild chronic inflammation	1
		Moderate mixed inflammation	2
		Severe acute inflammation	3
Collagen deposition	Assessed using Masson’s trichrome staining (0–3 scale)	Minimal collagen matrix	0
		Loose, immature collagen	1
		Moderate organized collagen	2
		Dense, mature collagen architecture	3

The time to achieve complete wound closure, defined as 100% epithelialization with no drainage, of the target ulcer was assessed by the end of six weeks. The proportion of patients to achieve complete closure was determined by the proportion of patients who achieved complete closure over the six-week treatment period. Next, the mean number of repeated applications of the HPTC and dHCAM used to obtain wound closure over six weeks was noted. Lastly, the incidence of adverse events related to the intervention, such as infection and allergic reactions, if any, was noted over the six-week study period.

Change in pain was measured using the Visual Analog Scale, with scores ranging from 0 to 10 (0 = “no pain” to 10 = “severe pain”) (time frame = seven weeks, including the one-week follow-up). Pain scores were assessed weekly. Change in the quality of life was also assessed, using the Wound-QoL questionnaire rated as “not at all,” “a little,” “moderately,” “quite a lot,” and “very much” for 17 questions (time frame = seven weeks, including the one-week follow-up). Finally, the healed wound appearance was assessed using the Manchester Scar Scale. The resultant new skin was assessed and documented at each visit using the Manchester Scar Scale, assessing color, finish, contour, distortion, and texture, with values ranging from 1 to 4 (1 = “excellent” and 4 = “poor”). This was done during the follow-up period.

Sample size calculation

The sample size was calculated using the standard two-sample comparison of proportions (e.g., wound closure rate), assuming an α of 0.05 (5%), power of 0.80 (80%), proportion of success in control (p₁) of 0.60 (typical healing rate for dHACM), and proportion of treatment success (p₂) of 0.90 (expected improvement with type I collagen). We used the following formula for comparing two proportions: n = [(Z_α/2_ + Z_β_)^2^ × (p_1_(1 - p_1_) + p_2_(1 - p_2_))] / (p_1_ - p_2_)^2^, where Z_α/2_ is 1.96 for an α of 0.05 and Z_β_ is 0.84 for 80% power. Hence, the sample size of 30 per group was considered to be statistically adequate to detect a difference from 60% to 90% healing rate with 80% power.

Randomization and blinding

Patients were randomized 1:1 to the HPTC or dHCAM group using a computer-generated randomization sequence following an open-label design (patients and clinicians were aware of the treatment allocation). Outcome assessors and statisticians were blinded to the treatment allocation.

Human subjects and ethical approval

This study was conducted in accordance with the ethical principles of the Declaration of Helsinki and Good Clinical Practice guidelines. Ethical clearance was obtained from the Institutional Ethics Committee of Adichunchanagiri Institute of Medical Sciences, Karnataka, India (approval number: AIMS/IEC/004/2025). All participants provided written informed consent before enrolment. The trial was prospectively registered with ClinicalTrials.gov (registration number: NCT06831760), and the registration details are publicly available. Patient confidentiality and data protection were maintained throughout the study.

Data collection and monitoring

Baseline data, such as demographics, medical history, and ulcer characteristics, were collected. Weekly follow-ups were performed to collect wound measurements (photographic documentation), assess healing progress, record pain scores, record adverse events, and document patient-reported outcomes. Histopathological specimens were obtained at baseline and day five for vascularity assessment. At the end-of-study visit at week six, i.e., final wound assessment, patient feedback on treatment experience, quality of life, and scar assessment was noted. Data entry and monitoring were managed using an electronic data capture system.

Data analysis

Data were analyzed using SPSS version 26.0 (IBM Corp., Armonk, NY, USA). Continuous variables were expressed as mean ± standard deviation, and categorical variables as frequencies and percentages. Student’s t-test was used for comparing continuous variables between groups, and the chi-square test was used for categorical variables. A p-value <0.05 was considered statistically significant. Kaplan-Meier survival analysis was used to assess the time to complete healing, and Cohen’s d values were used for primary histopathological parameters.

## Results

In this study, baseline demographics and ulcer characteristics were assessed. A total of 60 patients were enrolled and randomized, with 30 patients in each group. Baseline characteristics were well-balanced between groups (Table 2).

**Table 2:** Baseline demographics and ulcer characteristics. *: Statistically significant (p < 0.05).

Parameter	High-purity type I collagen-based skin substitute group (n = 30)	Dehydrated human amnion/chorion membrane group (n = 30)	P-value
Age (years), mean ± standard deviation	63.2 ± 10.8	66.1 ± 11.4	0.287
Gender (male/female)	20/10	14/16	0.082
Ulcer duration (months), mean ± standard deviation	2.1 ± 1.0	2.3 ± 1.3	0.512
Baseline ulcer size (cm²), mean ± standard deviation	14.8 ± 1.9	15.0 ± 1.8	0.645

The mean age was 63.2 ± 10.8 years in the HPTC group and 66.1 ± 11.4 years in the dHACM group. Gender distribution showed 66.67% males and 33.33% females in the HPTC group versus 46.67% males and 53.33% females in the dHACM group. The mean ulcer duration was 2.1 ± 1.0 months in the HPTC group and 2.3 ± 1.3 months in the dHACM group. Comorbidities such as hypertension and diabetes were evenly distributed. Wound duration ranged from one to four months in both groups, with no significant difference in baseline wound characteristics.

The primary outcome, namely, wound area reduction, was assessed. The HPTC group demonstrated superior wound area reduction (78.9 ± 17.8%) compared to the dHACM group (65.4 ± 7.9%) (p = 0.001) at seven weeks. Progressive wound area reduction was observed at all time points, with significant differences emerging by week four (p < 0.05) (Tables 3, 4).

**Table 3:** Ulcer size progression (cm²). *: Statistically significant (p < 0.05).

Time Point	High-purity type I collagen-based skin substitute group (mean ± SD)	Dehydrated human amnion/chorion membrane group (mean ± SD)	P-value
Baseline	14.8 ± 1.9	15.0 ± 1.8	0.645
Day 7	12.8 ± 2.3	13.0 ± 2.1	0.723
Day 14	10.7 ± 2.8	11.2 ± 2.4	0.456
Day 21	8.7 ± 3.2	9.6 ± 2.1	0.198
Day 28	6.1 ± 4.2	7.9 ± 2.3	0.045*
Day 35	4.2 ± 4.1	6.4 ± 2.5	0.012*
Day 42	2.8 ± 3.6	5.2 ± 2.1	0.002*

**Table 4:** Wound closure outcomes. *: Statistically significant (p < 0.05).

Outcome	High-purity type I collagen-based skin substitute group	Dehydrated human amnion/chorion membrane group	P-value
Mean % wound area reduction at 7 weeks	78.9 ± 17.8	65.4 ± 7.9	0.001*
Complete closure, n (%)	21 (70%)	13 (43.3%)	0.031*
Time to complete closure (days), mean ± standard deviation	42.6 ± 9.8	46.2 ± 8.7	0.047*

At seven weeks, complete wound closure was achieved in 21 (70%) patients in the HPTC group compared to 13 (43.3%) patients in the dHACM group (p = 0.031). The relative risk of healing was 1.62 (95% confidence interval (CI) = 1.02-2.57), favoring the HPTC group (Table 4). Among patients who achieved complete healing, the mean time to closure was significantly shorter in the HPTC group (42.6 ± 9.8 days) compared to the dHACM group (46.2 ± 8.7 days) (p = 0.047). Kaplan-Meier analysis demonstrated faster healing kinetics in the HPTC group throughout the study period (Table 4).

The histopathological analysis revealed statistically significant superiority of HPTC over dHCAM across all measured parameters on day five compared to the baseline on day zero (Table 5).

**Table 5:** Comparison of histopathological parameters (day 5). *: Statistically significant (p < 0.05).

Parameter	High-purity type I collagen-based skin substitute group (n = 30)	Dehydrated human amnion/chorion membrane group (n = 30)	P-value
Vascularity score	2.73 ± 0.45	1.87 ± 0.68	<0.001*
Neo-epithelialization score	2.67 ± 0.48	1.63 ± 0.72	<0.001*
Fibroblast activity score	2.80 ± 0.41	1.93 ± 0.64	<0.001*
Capillary density (vessels/mm²)	47.3 ± 8.2	28.7 ± 9.6	<0.001*
Inflammation score	1.23 ± 0.43	2.17 ± 0.59	<0.001*
Collagen deposition score	2.63 ± 0.49	1.77 ± 0.63	<0.001*

HPTC demonstrated 46% higher vascular infiltration scores (95% CI = 0.62-1.10; p < 0.001) (Table 6). The statistical analysis revealed a highly significant association between treatment groups and vascularity outcomes (χ² = 31.847, degrees of freedom = 3, p < 0.001), indicating that the observed differences were not due to chance. The strength of this association was further confirmed by Cramér’s V of 0.729, which represented a large effect size and demonstrated a substantial relationship between the type of treatment and vascular development. Most notably, the HPTC group demonstrated markedly superior vascularity compared to the dHACM group, with 76.7% of subjects achieving the highest grade of vascularity (Grade 3) versus only 6.7% in the dHACM group.

**Table 6:** Detailed vascularity assessment. *: Statistically significant (p < 0.05).

Vascularity grade	High-purity type I collagen-based skin substitute group, n (%)	Dehydrated human amnion/chorion membrane group, n (%)	Chi-square p-value
Grade 0	0 (0%)	3 (10%)	<0.001*
Grade 1	1 (3.3%)	12 (40%)	
Grade 2	6 (20%)	13 (43.3%)	
Grade 3	23 (76.7%)	2 (6.7%)	

HPTC also showed 64% superior epithelial migration (95% CI = 0.71-1.37; p < 0.001) (Table 7). The chi-square analysis revealed a statistically significant association between treatment groups and epithelial migration outcomes (χ² = 28.974, df = 3, p < 0.001), indicating that the observed differences in healing patterns were not due to chance. The substantial effect size, as measured by Cramér’s V of 0.695, represented a large practical significance, reflecting dramatic distribution differences between the treatment groups. Most notably, the HPTC treatment demonstrated markedly superior therapeutic efficacy, with 73.3% of cases achieving Grade 3 epithelial migration compared to only 6.7% in the dHACM group.

**Table 7:** Neo-epithelialization distribution. *: Statistically significant (p < 0.05).

Migration grade	High-purity type I collagen-based skin substitute group, n (%)	Dehydrated human amnion/chorion membrane group, n (%)	Chi-square p-value
Grade 0	0 (0%)	4 (13.3%)	<0.001*
Grade 1	2 (6.7%)	11 (36.7%)	
Grade 2	6 (20%)	13 (43.3%)	
Grade 3	22 (73.3%)	2 (6.7%)	

HPTC exhibited 45% higher fibroblast activity (95% CI = 0.58-1.16; p < 0.001) (Table 8). This analysis revealed a statistically significant association between the treatment groups and fibroblast activity outcomes. The chi-square test yielded a value of 33.562 with three degrees of freedom, with a p-value less than 0.001, indicating that the observed differences between groups were highly unlikely to have occurred by chance alone. The effect size, measured by Cramér’s V of 0.748, demonstrated a large practical significance, reflecting the dramatic contrast in Grade 3 achievement rates between the treatment conditions. Most notably, the HPTC group demonstrated markedly superior fibroblast activity, with 83.4% of cases achieving Grade 3 performance compared to only 6.7% in the dHACM group.

**Table 8:** Fibroblast activity analysis. *: Statistically significant (p < 0.05).

Activity level	High-purity type I collagen-based skin substitute group, n (%)	Dehydrated human amnion/chorion membrane group, n (%)	Chi-square p-value
Grade 0	0 (0%)	2 (6.7%)	<0.001*
Grade 1	1 (3.3%)	10 (33.3%)	
Grade 2	4 (13.3%)	16 (53.3%)	
Grade 3	25 (83.4%)	2 (6.7%)	

HPTC demonstrated 65% higher capillary density (95% CI = 13.2-24.0 vessels/mm²; p < 0.001). HPTC showed 43% lower inflammatory scores (95% CI = -1.19 to -0.69; p < 0.001) (Table 9). The chi-square analysis revealed a statistically significant association between treatment groups and inflammation levels (χ² = 22.891, df = 3, p < 0.001), indicating that the observed differences in inflammation outcomes across groups were highly unlikely to have occurred by chance alone. The large effect size, as measured by Cramér’s V of 0.618, demonstrated a substantial practical significance of this association, with the pattern notably favoring lower inflammation levels in the HPTC group compared to the dHACM group. Additionally, the HPTC treatment demonstrated remarkable efficacy in promoting tissue repair, achieving 49% superior collagen organization compared to the dHACM group. This improvement in collagen deposition was highly statistically significant (95% CI = 0.56-1.16; p < 0.001), indicating both the reliability and magnitude of the therapeutic benefit. HPTC demonstrated better inflammation control with 26.7% showing Grade 0 (no inflammation) versus 3.3% in the dHACM group

**Table 9:** Inflammation pattern comparison. *: Statistically significant (p < 0.05).

Inflammation grade	High-purity type I collagen-based skin substitute group, n (%)	Dehydrated human amnion/chorion membrane group, n (%)	Chi-square p-value
Grade 0	8 (26.7%)	1 (3.3%)	<0.001*
Grade 1	19 (63.3%)	7 (23.3%)	
Grade 2	3 (10%)	18 (60%)	
Grade 3	0 (0%)	4 (13.4%)	

All measures demonstrated highly significant results (p < 0.001 for all comparisons), large effect sizes based on the distribution patterns, and consistent superiority of the HPTC-based skin substitute across all healing parameters. The chi-square tests revealed statistically significant and clinically meaningful differences favoring the HPTC treatment across vascularity, epithelialization, fibroblast activity, and inflammation control in VLU healing.

The effect size analysis using Cohen’s d values revealed substantial and clinically meaningful differences in primary histopathological parameters between treatment groups. The HPTC treatment demonstrated large positive effects across multiple healing indicators, with vascularity showing a Cohen’s d of 1.45, neo-epithelialization at 1.71, fibroblast activity at 1.58, and collagen deposition at 1.51, all representing large effect sizes that indicated robust therapeutic benefits. Most notably, capillary density exhibited a very large effect size of 2.12, suggesting exceptional improvement in microvascular formation and tissue perfusion. The inflammation parameter showed a large negative effect size of -1.82, favoring the HPTC group, indicating significantly reduced inflammatory response compared to the dHACM group.

No serious adverse events were reported in either group. Minor adverse events included mild local irritation in two (6.7%) patients in the HPTC group and three (10%) patients in the dHACM group (p = 0.640). All adverse events resolved spontaneously without intervention.

Both groups showed a reduction in pain scores over time, from baseline to seven weeks, with no significant difference between groups. Between-group comparison showed a trend toward better pain control in the HPTC group (p = 0.074) (Table 10).

**Table 10:** Pain score progression.

Week	High-purity type I collagen-based skin substitute group (mean ± SD)	Dehydrated human amnion/chorion membrane group (mean ± SD)	P-value
Week 1	2.9 ± 1.5	3.2 ± 1.6	0.456
Week 2	3.7 ± 1.3	3.4 ± 1.4	0.378
Week 3	2.8 ± 1.5	3.0 ± 1.3	0.587
Week 4	2.8 ± 1.5	3.2 ± 1.5	0.289
Week 5	2.7 ± 1.4	2.8 ± 1.2	0.756
Week 6	2.8 ± 1.6	3.0 ± 1.4	0.612
Week 7	2.9 ± 1.3	2.8 ± 1.5	0.823

Reapplication was required in eight (26.7%) patients in the HPTC group and 12 (40.0%) patients in the dHACM group (p = 0.263). During the follow-up period, no recurrence occurred in the HPTC group, but three (23.1%) patients in the dHACM group, among those who achieved complete healing, had recurrence (p = 0.0441). Quality of life improvement was observed in 70% of HPTC patients versus 40% of dHACM patients. The between-group difference was statistically significant toward better quality of life in the HPTC group (p = 0.025).

Using the Manchester Scar Scale, the structural stability of the scar was assessed as good in 19 (63.3%) patients in the HPTC group and 11 (36.7%) patients in the dHACM group, with fair stability in 10 (33.3%) patients and 14 (46.7%) patients, respectively, and poor quality in one (3.3%) patient in the HPTC group versus five (16.7%) patients in the dHACM group. The Manchester Scar Scale scores seemingly favored the HPTC group, but the finding was not statistically significant (p = 0.0649).

## Discussion

In this randomized controlled study, HPTC demonstrated superior efficacy compared to dHACM in promoting faster and more complete healing in VLUs. These findings mirror previous research in diabetic foot ulcers, where HPTC showed superior healing efficacy [21]. This study is unique as it is the first randomized trial to evaluate the efficacy of HPTC as a treatment for VLUs. It is also unique in its use of an explanatory endpoint to measure the results of healing with an advanced wound care product. Our results show that VLUs treated with HPTC heal in a significantly more rapid fashion than those treated with dHCAM alone. The findings have significant clinical implications for wound care practitioners and patients suffering from these challenging chronic wounds.

**Figure 1:**
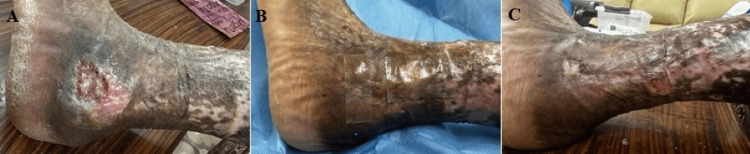
Percentage wound size reduction with the application of Helicoll®. Chronic venous ulcer on the lateral side of the left leg with a wound area of 24 cm² (A). Helicoll® application over the cleaned ulcer (B). Ulcer size at the end of seven weeks after Helicoll® application, with a significant percentage reduction in size of 90% (C).

The clinical significance of our findings extends beyond mere wound closure rates. The faster healing time observed with HPTC (42.6 ± 9.8 days vs. 46.2 ± 8.7 days) translates to reduced healthcare costs, decreased patient suffering, and improved quality of life. The progressive reduction in ulcer size was observed in both groups, with the HPTC group (6.1 ± 4.2 vs. 7.9 ± 2.3) showing statistically significant (p = 0.045) advantages from day 28 onwards. In our study, the HPTC group demonstrated superior wound area reduction (78.9 ± 17.8%) compared to the dHACM group (65.4 ± 7.9%) (p = 0.001) at seven weeks (Figure 1). This delayed but sustained effect suggests that HPTC may provide more robust long-term healing benefits. The accelerated healing pattern observed with HPTC is consistent with its collagen matrix properties that support angiogenesis and tissue remodelling [23,24].

The statistically significant (p = 0.031) higher complete wound closure rate in the HPTC group (70% vs. 43.3%) suggests that HPTC may provide superior wound healing properties compared to dHACM. This finding is consistent with previous studies that have demonstrated the effectiveness of collagen-based skin substitutes in promoting tissue regeneration [25,26]. The bioactive properties of HPTC, including its ability to provide a scaffold for cellular migration and proliferation, may contribute to enhanced wound healing outcomes [27]. The consistent superiority of HPTC across different wound sizes and durations suggests that this treatment modality may be beneficial for a wide range of patients with VLUs.

The comprehensive histopathological analysis at day five post-treatment compared to baseline provides compelling microscopic evidence supporting the clinical superiority of HPTC over dHCAM in VLU management. The statistically significant improvements across all measured parameters demonstrate the multifaceted advantages of type I collagen-based wound therapy.

The remarkable 46% improvement in vascularity scores (2.73 vs. 1.87, p < 0.001) with HPTC treatment reflects superior angiogenic stimulation. The capillary density analysis further corroborates this finding, with HPTC demonstrating 65% higher vessel density (47.3 vs. 28.7 vessels/mm², p < 0.001). This enhanced vascularization is crucial for wound healing as it ensures adequate oxygen and nutrient delivery to healing tissues. The large effect size (Cohen’s d = 2.12) for capillary density represents one of the most substantial treatment differences observed, suggesting that HPTC’s collagen matrix provides superior scaffolding for endothelial cell migration and vessel formation.

The 64% superior neo-epithelialization scores in the HPTC group (2.67 vs. 1.63, p < 0.001) indicate accelerated wound closure mechanisms. Notably, 73.3% of HPTC-treated wounds achieved Grade 3 epithelialization compared to only 6.7% in the dHCAM group. This dramatic difference suggests that HPTC’s native collagen structure provides optimal conditions for keratinocyte migration and proliferation. The absence of Grade 0 epithelialization in the HPTC group versus 13.3% in the dHCAM group further emphasizes the consistent healing response achieved with collagen-based therapy.

The 45% higher fibroblast activity scores (2.80 vs. 1.93, p < 0.001) demonstrate HPTC’s superior ability to stimulate cellular wound healing responses. With 83.4% of HPTC specimens showing Grade 3 fibroblast activity versus only 6.7% in the dHCAM group, the data indicate robust cellular activation. This enhanced fibroblast response is critical for extracellular matrix synthesis and wound strength development. The type I collagen substrate appears to provide optimal biochemical signals for fibroblast chemotaxis and activation.

Perhaps most significantly, HPTC demonstrated superior inflammation control with 43% lower inflammatory scores (1.23 vs. 2.17, p < 0.001). The absence of Grade 3 inflammation in HPTC-treated wounds compared to 13.4% in the dHCAM group suggests better biocompatibility and reduced tissue reactivity. This controlled inflammatory environment is crucial for optimal healing, as excessive inflammation can impede wound closure and increase complications.

The 49% superior collagen deposition scores (2.63 vs. 1.77, p < 0.001) indicate accelerated and improved matrix remodelling. The organized collagen architecture observed in HPTC-treated wounds suggests better long-term wound strength and reduced recurrence risk as a result of enhanced structural integrity and long-term durability. This finding aligns with the known benefits of providing native collagen scaffolding for tissue regeneration, resulting in stronger, more organized scar tissue with improved biomechanical properties.

The greater improvement in histopathological parameters in the HPTC group indicates enhanced tissue regeneration and vascularization. This finding supports the hypothesis that HPTC’s bioactive components facilitate better tissue integration and healing compared to dHACM [28]. The improved vascularity observed in the HPTC group correlates with clinical healing outcomes, suggesting a mechanistic basis for the observed therapeutic benefits.

The better pain reduction, though not statistically significant, observed in the HPTC group is particularly important, as chronic pain is a major concern for patients with VLUs. The pain reduction observed in both groups indicates that both HPTC and dHACM are effective in managing ulcer-related discomfort [29,30].

While both treatments showed improvements in quality of life measures, the difference was statistically significant toward better quality of life in the HPTC group (p = 0.025). This finding suggests that both treatments provide meaningful clinical benefits from the patient’s perspective.

The absence of serious adverse events in both groups demonstrates the safety of both treatments. Minor adverse events included mild local irritation in two (6.7%) patients in the HTPC group and three (10%) patients in the dHACM group (p = 0.640).

Although no recurrence was noted in the HPTC group, three (23.1%) patients in the dHACM group who achieved complete healing showed recurrence (p = 0.0441). This safety profile is consistent with previous reports of collagen-based treatments [31-33]. The reduced need for reapplication in the HPTC group (26.7% vs. 40.0%) may translate to reduced healthcare costs and improved patient compliance, though this difference was not statistically significant.

Structural stability of the scar was assessed as good/fair in 29 (96.6%) patients in the HPTC group, while 25 (83.3%) patients demonstrated the same in the dHACM group. The Manchester Scar Scale seemingly favored the HPTC group, though this was not statistically significant (p = 0.0649).

Sood et al. showed improved vascularization and faster healing with HPTC compared to standard dressings [34]. Similarly, a study by Gunasekaran demonstrated that HPTC-treated wounds showed faster granulation and epithelialization compared to dHACM, supporting the efficacy of collagen substitutes. This dual-site study in India and the United States further underscored the reproducibility of results across different healthcare systems [14]. The role of type I collagen in wound healing is well documented. It provides structural integrity, modulates inflammation, and promotes fibroblast migration [11]. A randomized trial by Narayan et al. showed accelerated healing in collagen-treated diabetic ulcers compared to controls [13].

The ultimate goal of treating VLUs is to achieve complete healing, yet fewer than two-thirds (62%) of all VLUs heal by 24 weeks with standard care [35]. Given the long healing period, intermediate endpoints such as percentage change in wound area by the fourth week of treatment have been shown to be important surrogate markers of complete wound healing by 12 or 24 weeks [36].

Previous studies have reported variable success rates with different advanced wound care products. A systematic review by Shu et al. [37] reported complete healing rates of 40-60% for collagen-based dressings, which aligns with our findings for HPTC. Similarly, studies on amniotic membrane products have shown healing rates of 20-40% [38,39], consistent with our dHACM results. Another study by Lavery et al. [21] confirmed the superior performance of HPTC in diabetic foot ulcers compared to dHACM, reinforcing the present findings in the context of VLUs.

A preclinical study by Bush et al. on bovine dermal collagen matrix supported HPTC’s vascular regenerative capabilities and integration into host tissues [23]. Further, a recent observational study by Dhanraj et al. found HPTC to be effective in covering bare tendons and bones where flap coverage was not feasible [20]. Our findings align with previous studies demonstrating the effectiveness of collagen-based skin substitutes in hard-to-heal VLUs.

Of note, venous ulcers are known to mostly resolve with the treatment of underlying chronic venous insufficiency. In all our patients, no specific surgical intervention was employed, and only the conservative standard compression therapy was instituted with the use of advanced skin dressings.

dHACM, although rich in growth factors and anti-inflammatory agents, may have lower mechanical durability and slower bioreabsorption than type I collagen. Unlike HPTC, dHACM’s potential immunogenicity remains a concern. Additionally, scar improvement and patient comfort were superior in the HPTC group.

The HPTC used in this study is a commercially available skin substitute, namely, Helicoll®, manufactured by Encoll Corp. This HPTC is made of high-purity (>97%) type I collagen via the US-patented twice treatment process to purify the protein. As type I collagen is the least immunogenic protein (due to its highest homology between other type I collagens of all recognized species), it retains a high degree of biocompatibility when used as a skin substitute. Additionally, the particular commercial-grade HPTC is further bioactivated by a patented phosphorylation process. Accordingly, Helicoll® is clinically proven to attract the cells (through cell signal transduction), resulting in the formation of new blood capillaries within four to five days of application [40].

Potential reasons for the superior clinical outcomes observed with Helicoll® are the exceptional purity of type I collagen, wherein Helicoll® employs a patented formulation of highly purified type I collagen, with documented standards of purity, which may enhance biocompatibility and minimize immunogenic response. Second, preservation of native collagen structure by employing a sterilization process utilizing ethylene oxide, which is scientifically recognized for preserving the native molecular conformation of collagen. In contrast, methods such as electron beam, gamma irradiation, or other high-energy sterilization techniques are known to compromise the structural integrity of type I collagen, potentially impairing its biological functionality. Third, biomimetic structural configuration, i.e., the physical architecture of Helicoll®, closely mimics the natural dermal matrix. It features a collagen fiber arrangement analogous to that of the human skin, with an optimal porosity of approximately 20 µ. This structural fidelity promotes effective cellular infiltration and tissue integration. Conversely, some other collagen-based dressings exhibit a sponge-like morphology with larger pore sizes (200-400 µ), which may hinder optimal cell migration and proliferation. Lastly, bioactivation via phosphorylation, wherein, beyond its purity, Helicoll® incorporates a proprietary bioactivation process involving the phosphorylation of type I collagen. This biochemical modification enhances cell signaling pathways, facilitating the recruitment of reparative cells, including somatic stem cells, to the wound site, thereby promoting tissue repair and regeneration. Type I collagen provides an optimal biological scaffold that closely mimics the natural extracellular matrix, facilitating cellular migration, proliferation, and angiogenesis. The high-purity collagen structure maintains bioactivity while providing hemostatic properties that help establish a stable wound environment.

The other collagen preparations, mostly contaminated with type III collagen, elastin, lipids, and other immunogenic proteins, following chemical cross-linking, which is required to minimize their immunogenicity, do not maintain the native chemistry of collagen and thereby lose their bioactivity. Moreover, other binding abilities get significantly impaired with its wound healing abilities.

Collagen-based scaffolds such as Helicoll® provide a biologically active matrix promoting fibroblast migration, neovascularization, and epithelialization [41]. dHACM also contains growth factors but may elicit more inflammation, as seen in our study, potentially explaining the slower healing observed [42]. The higher rate of complete closure and better scar quality seen in Helicoll® could be due to its higher structural stability and modulation of vascularity, as noted in histopathological findings.

Another salient feature of HPTC to help heal VLUs is the glycosylation of lysine residues of type I collagen in hyperglycemic patients. As a result, the conversion of amino groups to aldehyde groups is impeded, leading to a disruption in the aldol-condensation reaction necessary for the regular maturation of wound bed collagen for the proper healing of the wound. The local administration HPTC for VLUs would absorb excess glucose by serving as a substrate for free-floating glucose/glycans, thereby reducing the unwanted impact of glucose on the wound-bed collagen [43].

A strength of our study is its randomized controlled design, although caregivers were unable to be blinded to group assignment. Standardized treatment protocols were implemented. Objective measurements were used to determine wound size, and a comprehensive outcome assessment was conducted in a specific sequence during the study to reduce bias. Operationally, a shorter study duration reduces the risk for non-compliance, loss to follow-up, and missing data, resulting in more accurate observations.

Limitations

Several limitations of this study should be acknowledged. First, the single-center design may limit generalizability to other healthcare settings and populations. Second, the relatively short follow-up period (seven weeks), while sufficient for assessing acute healing outcomes, may not capture long-term outcomes, recurrence rates, or durability of treatment effects. Third, the study was not completely blinded due to the nature of the interventions, which may have introduced bias in subjective assessments. Fourth, the modest sample size, while adequate for the primary endpoint for detecting significant differences, may be insufficient for detecting differences in secondary outcomes and rare adverse events. A larger sample size would provide greater statistical power and allow for more robust analyses. The study included a specific range of wound sizes and may not be generalizable to very large wounds.

Future studies should include multi-center designs with longer follow-up periods to assess the durability of healing and recurrence rates. Cost-effectiveness analyses comparing both treatments would provide valuable information for healthcare decision-making. Additionally, mechanistic studies investigating the molecular pathways involved in healing with different skin substitutes to understand the biological basis for the observed differences would enhance understanding of optimal treatment selection. Further, there is a need to determine the ideal frequency of HPTC application. However, the results of this first clinical trial support the use of HPTC as an efficacious treatment for VLUs.

Clinical implications

The findings of this study have important clinical implications for the management of VLUs. The superior efficacy of HPTC suggests that type I collagen-based skin substitutes should be considered first-line advanced wound care therapy for chronic VLUs. The superior cellular responses, enhanced vascularization, accelerated re-epithelialization, optimal inflammatory modulation, and superior extracellular matrix remodelling collectively contribute to faster wound healing, reduced infection risk, and better long-term outcomes, with improved patient satisfaction scores. The consistency of findings across all parameters strengthens the evidence for HPTC therapeutic superiority. The faster healing times and improved quality of life outcomes support the clinical value of this approach. Healthcare providers should consider patient-specific factors when selecting between advanced wound care modalities. The standardized composition and manufacturing process of HPTC may provide more predictable outcomes compared to biologically variable products. This study aims to provide high-quality evidence for clinicians to select the most effective treatment for VLUs, improving outcomes and patient quality of life.

## Conclusions

This randomized controlled trial demonstrated that HPTC (Helicoll®) is superior to dHACM in achieving complete wound closure and reducing healing time in VLUs. The excellent complete healing rate achieved with HPTC represents a clinically meaningful improvement over current treatment options. The superior healing kinetics observed with Helicoll® suggest that this treatment may offer better long-term healing benefits for patients with VLUs. These findings suggest that Helicoll® represents a promising therapeutic option for chronic VLU management, offering faster healing by higher complete wound closure rates, faster healing times, and greater wound area reduction. Complementing this are reduced pain, reduced complications, better scar outcomes, lower recurrence, improved quality of life, and potentially reduced healthcare costs. The comprehensive histopathological analysis at day five post-application showed statistically significant improvements in vascularity infiltration, neo-epithelialization, fibroblast activity, capillary density, optimal inflammatory modulation, and superior collagen deposition, collectively demonstrating that Helicoll® promotes superior wound healing through multiple complementary mechanisms. These objective histological findings, evaluated by blinded pathologists, provide robust scientific evidence supporting HPTC. The large effect sizes observed across all parameters indicate clinically meaningful differences that translate to improved patient outcomes. Further research is needed to confirm these findings in larger, multi-center trials with longer follow-up periods to provide more robust evidence for clinical practice guidelines. Cost-effectiveness analyses and mechanistic studies would provide additional insights into the optimal use of advanced wound care technologies in clinical practice. The results of this study contribute to the growing body of evidence supporting the use of advanced wound care products in VLU management and provide clinicians with valuable data to guide treatment decisions in this challenging patient population. Healthcare providers should consider incorporating collagen-based skin substitutes into their treatment algorithms for VLUs, particularly in patients with refractory wounds or those requiring faster healing.
